# Drug-associated hyperammonaemia: a Bayesian analysis of the WHO Pharmacovigilance Database

**DOI:** 10.1186/s13613-022-01026-4

**Published:** 2022-06-18

**Authors:** Alexander Balcerac, Kevin Bihan, Bénédicte Lebrun-Vignes, Dominique Thabut, Joe-Elie Salem, Nicolas Weiss

**Affiliations:** 1 Département de neurologie, Unité de Médecine Intensive Réanimation À Orientation Neurologique, Sorbonne Université, AP-HP.Sorbonne Université, Hôpital de La Pitié-Salpêtrière, 47-83, boulevard de l’hôpital, 75013 Paris, France; 2 Brain Liver Pitié-Salpêtrière (BLIPS) Study Group, INSERM UMR_S 938, Centre de Recherche Saint-Antoine, Maladies métaboliques, biliaires et fibro-inflammatoire du foie, Institute of Cardiometabolism and Nutrition (ICAN), Paris, France; 3 Groupe de Recherche Clinique en REanimation Et Soins Intensifs du Patient en Insuffisance Respiratoire aiguE (GRC-RESPIRE), Sorbonne Université, Paris, France; 4 Department of Pharmacology, Regional Pharmacovigilance Center, Sorbonne Université, AP-HP.Sorbonne Université, Pitié-Salpêtrière Hospital, INSERM, Sorbonne Université, Paris, France; 5 Sorbonne Université, AP-HP.Sorbonne Université, Hôpital de La Pitié-Salpêtrière, service d’hépatogastroentérologie, Unité de Soins Intensifs d’hépatologie, Paris, France

## Abstract

**Background:**

Hyperammonaemia is frequent in Intensive Care Unit patients. Some drugs have been described as associated with this condition, but there are no large-scale studies investigating this topic and most descriptions only consist of case-reports.

**Methods:**

We performed a disproportionality analysis using VigiBase, the World Health Organization Pharmacovigilance Database, using the information component (IC). The IC compares observed and expected values to find associations between drugs and hyperammonaemia using disproportionate Bayesian reporting. An IC_0.25_ (lower end of the IC 95% credibility interval) > 0 is considered statistically significant. The main demographic and clinical features, confounding factors, and severity of cases have been recorded.

**Results:**

We identified 71 drugs with a disproportionate reporting in 2924 cases of hyperammonaemia. Most of the suspected drugs could be categorised into 4 main therapeutic classes: oncologic drugs, anti-epileptic drugs, immunosuppressants and psychiatric drugs. The drugs most frequently involved were valproic acid, fluorouracil, topiramate, oxaliplatin and asparaginase. In addition to these molecules known to be responsible for hyperammonaemia, our study reported 60 drugs not previously identified as responsible for hyperammonaemia. These include recently marketed molecules including anti-epileptics such as cannabidiol, immunosuppressants such as basiliximab, and anti-angiogenics agents such as tyrosine kinase inhibitors (sunitinib, sorafenib, regorafenib, lenvatinib) and monoclonal antibodies (bevacizumab, ramucirumab). The severity of cases varies depending on the drug class involved and high mortality rates are present when hyperammonaemia occurs in patients receiving immunosuppressant and oncologic drugs.

**Conclusions:**

This study constitutes the first large-scale study on drug-associated hyperammonaemia. This description may prove useful for clinicians in patients’ care as well as for trial design.

**Graphical Abstract:**

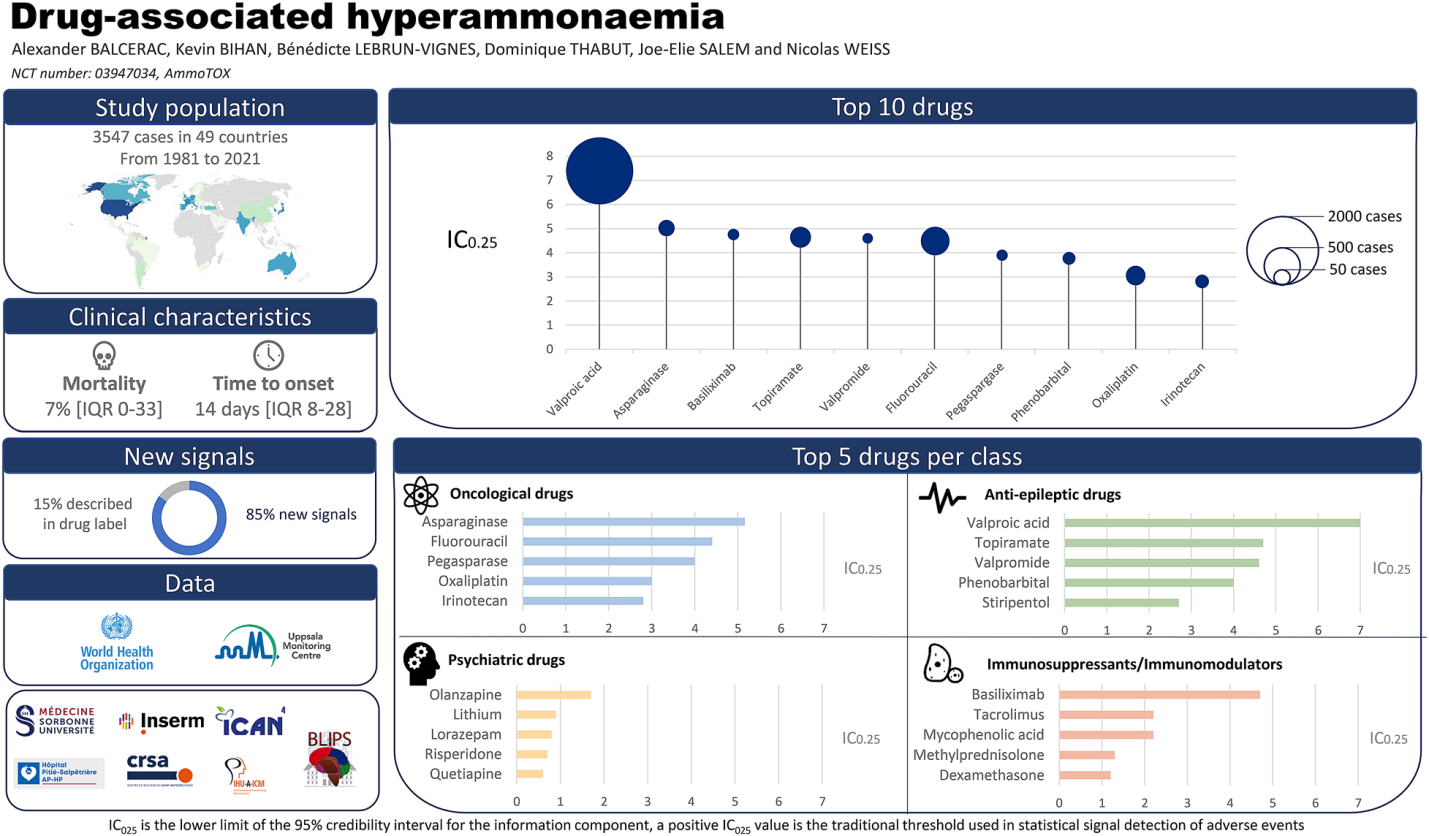

**Supplementary Information:**

The online version contains supplementary material available at 10.1186/s13613-022-01026-4.

## Introduction

Patients with hyperammonaemia frequently need management in the intensive care unit (ICU) or by intensivists. Hyperammonaemia is estimated to be present in about 4% of ICU patients [1]. Its real prevalence is, however, thought to be greater since some symptoms are non-specific: delirium, seizures, coma, brain oedema or brain herniation [2]. Acute liver failure or acute-on-chronic liver failure are the most prevalent causes of hyperammonaemia [1], but other less frequent actionable causes have to be suspected, in the absence of liver disease and maybe even in its presence: porto-systemic shunts without liver disease, inborn errors of metabolism, infections, some specific surgical procedures and drug-associated hyperammonaemia [3–10]. Acute hyperammonaemia is associated with a significant morbidity and mortality [11] that could be as high as 30 to 50% in “non-hepatic” hyperammonaemia [12]. Thus, in the most dramatic cases, brain oedema occurs and can lead to rapid herniation and death, whereas in less severe cases, prolonged hyperammonaemia can be associated to neurological sequelae. An urgent management is therefore mandatory in order to identify hyperammonaemia and to prevent its unfavourable evolution, by using either ammonia-lowering agents aimed to eliminate ammonia [13], extrarenal replacement therapy, or substances that reduce its production or absorption (oral disaccharides or polyethylene glycol). Several drugs have been described in the literature as responsible for hyperammonaemia, such as valproic acid [14, 15], L-asparaginase [16], fluorouracil [17] or capecitabine [18]. However, there are no large-scale studies investigating the drugs responsible for hyperammonaemia and most descriptions only consist of case-reports. The aim of the study was to identify drugs associated with the occurrence of hyperammonaemia using the WHO Global Pharmacovigilance Database.

## Methods

### VigiBase

Data were extracted from VigiBase, the WHO Global Pharmacovigilance Database of individual case safety reports (i.e. cases hereafter). This database gathers pharmacovigilance information from over 130 countries [19]. Depending on the country, the source of reporting might be varied: pharmacovigilance specialists, healthcare professionals, marketing authorisation holders or in some cases patients themselves.

### Selection of cases

We have included all reported cases in VigiBase from inception in 1967 to November 29, 2020, associated with the following preferred terms (PT) related to hyperammonaemia: “hyperammonaemia”, and/or “hyperammonaemic encephalopathy” and/or “hyperammonaemic crisis”, using the Medical Dictionary for Drug Regulatory Activities (MedDRA 24.0). Only drugs labelled as suspect or interacting were analysed. Drugs for which all cases of hyperammonaemia were reported in a single country and those used to treat hyperammonaemia (= protopathic bias) were excluded.

### Causality assessment

A case–non-case analysis was performed for each drug in the database, using the information component (IC). Briefly, the IC is an indicator value for disproportionate reporting when using the Bayesian method for disproportionality analysis developed by Uppsala Monitoring Center [20]. This method compares the proportion of a given adverse event associated with a drug to the proportion of the same adverse event for all other treatments in VigiBase [20]. We deliberately chose to use information component (IC) as the disproportionality assessment method, which is more conservative than the reporting odds ratio (ROR), in order to avoid false-positive signals and strengthen the results.

The information component is obtained by the formula:

$$ {\text{IC}} = \log 2\left[ {\frac{{{\text{N}}observed + 0.5}}{{\frac{{{\text{N}}drug \times {\text{N}}effect}}{{{\text{N}}total}} + 0.5}}} \right], $$

where Nobserved is the number of reported cases for a given adverse drug reaction; Ndrug is the number of cases for a given drug, regardless of the adverse reaction reported; Neffect corresponds to the number of cases of a given adverse reaction, whatever the responsible drug; and Ntotal corresponds to the total number of cases in the database, regardless of the drug or adverse reaction.

IC_025_ is the lower limit of the 95% credibility interval for the information component. A positive IC_025_ value (IC_025_ > 0) is the threshold deemed significant and used in statistical signal detection [21–23]. Thus, an IC_025_ > 0 indicates a statistical association between a drug and the adverse event.

Finally, the informativity score and extrinsic imputability were assessed in each case and for each drug, as defined by the unique 2011 French Pharmacovigilance criteria [24]. Informativity score of cases (defined as NI per convention) was computed as follow: NI2 when both the time to onset and discontinuation of the drug is described, NI1 when only one of those characteristics is present and NI0 when neither are described. Extrinsic imputability of drugs (defined as B per convention) was computed as follow: B4 when the effect is expected (described in the summary of product characteristics); B3 when the effect is widely published with this drug in reference databases and/or books; B2 when cases are published in a scientific journal or in a database; B1 when the effect is not published. The summary of product characteristic for each drug was extracted from the European Medicines Agency database (https://www.ema.europa.eu/en/medicines) and from all three official FDA drug databases, as of February 01, 2021: Drugs@FDA (https://www.accessdata.fda.gov/scripts/cder/daf/), the FDA Online Label Repository (https://labels.fda.gov/ingredientname.cfm) and DailyMed (http://dailymed.nlm.nih.gov/dailymed/).

### Clinical characteristics, presentation, and outcome

Age, sex, the geographical origin of the report and time to onset were retrieved for each individual case. Time to onset was defined as the time between the first administration of the drug and the occurrence of the adverse event.

Based on the concomitant MedDRA terms co-reported in each case, we have classified the clinical features associated with cases of hyperammonaemia into five main categories: coma/altered consciousness, brain oedema, seizures, neuropsychiatric presentation, and miscellaneous neurological signs. To assess underlying liver and kidney function, we also studied for MedDRA terms suggesting these conditions, concomitantly reported in all cases. See Additional file 1: Table S1 for details.

Each report of hyperammonaemia was analysed to ascertain severity. For this we used the previously defined categories in VigiBase: death, life threatening, caused prolonged hospitalisation, disabling/incapacitating, congenital/birth defect and other medically important condition (Additional file 2: Table S2). Seriousness was defined as belonging at least to one of those categories.

### Statistics and management of missing data

Quantitative values are given as means or median, minimum and maximum, inter-quartile range (IQR). Qualitative values are given as numbers and percentage of the group they are issued. As previously stated, a IC_025_ > 0 was considered as significant and indicating a statistical association between the adverse event and the drug.

Among the cases analysed, the following characteristics contain missing data: the age at onset, sex of the patient and the time to onset. Detailed analysis was only carried out on the available data. The percentage of missing data for each characteristic and each drug is detailed in Additional file 2: Table S2.

## Results

### Drug selection

Between inception of VigiBase and November 29th, 2020, we identified 3547 cases of drug-associated hyperammonaemia. Among more than 20 000 drugs registered (WHO Drug active ingredient variants), 642 were associated with at least one case of hyperammonaemia. After first excluding the drugs with a non-significant IC_025_ (555 drugs), we excluded those reported from a single country (11 drugs), and those used to treat hyperammonaemia or conditions responsible for hyperammonaemia (5 drugs with probable protopathic bias: lactulose, rifaximin, sodium phenylbutyrate, benzoic acid, carglumic acid). We kept for further analysis 71 drugs among 642 (11%) with a significant association (IC_025_ > 0), involved in 2924 cases of hyperammonaemia between January 1, 1981 (date of the first reported case), and November 29, 2020.

### General characteristics

**Fig. 1:**
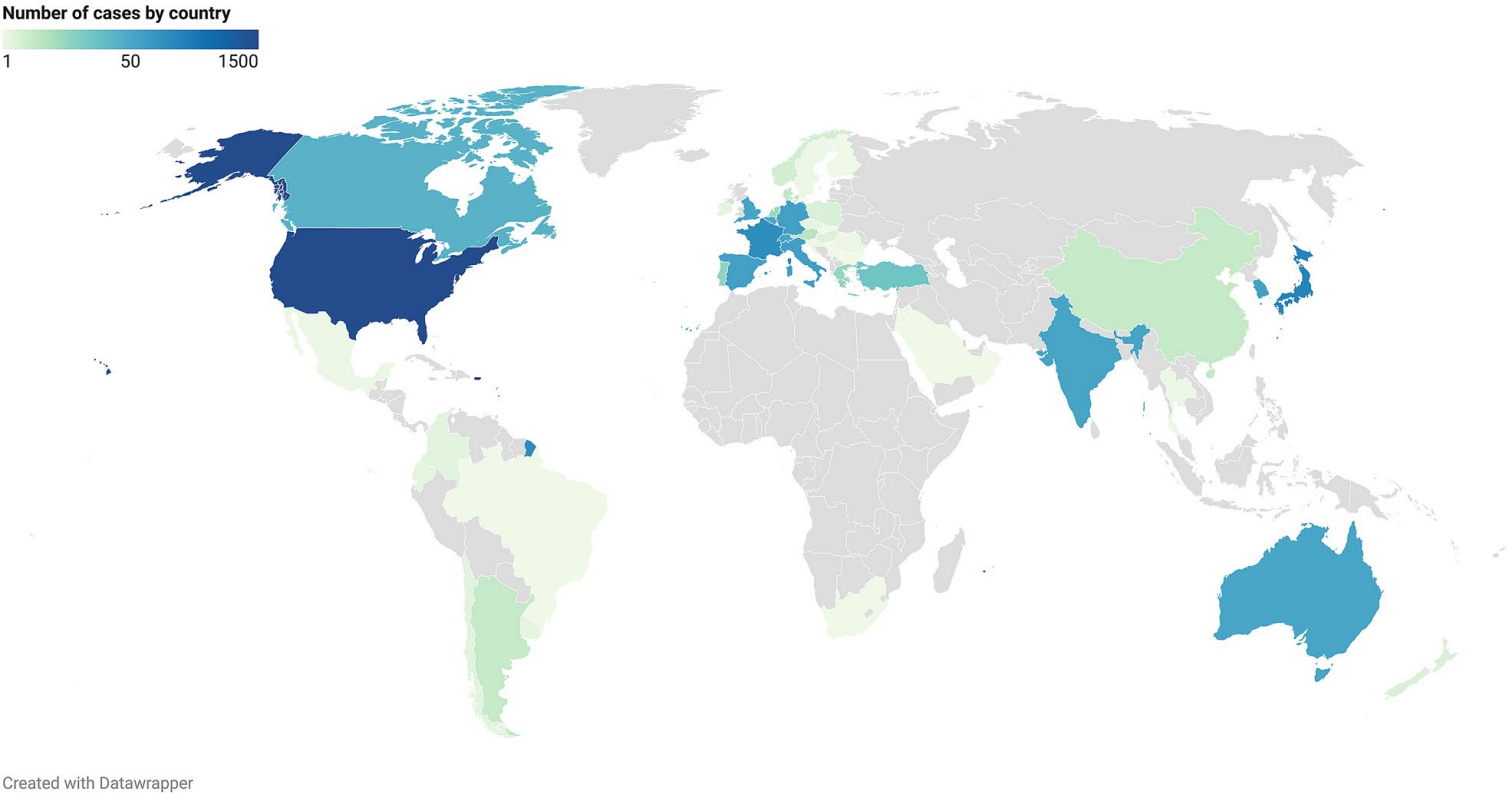
Number of reported cases according to each country. The non-linear scale displayed above indicates the colour corresponding to the number of cases reported for each country

The geographic origin of the cases is shown in Fig. 1: 40% were reported from the American continent, 34% from Europe, 22.5% from Asia, 2% from Oceania, 1% from the Middle East and 0.5% from Africa. In our study, 1% of cases were reported between 1981 and 1990, 6% between 1991 and 2000, 19% between 2001 and 2010 and 74% between 2011 and 2020 (Additional file 4: Fig. S1). Mean age of reported cases was 42.7 (standard deviation: 23.5 years; maximal range: 0–93 years), 54.5% of patients were male (*N* = 1 593) and hyperammonaemia occurred after a median time to onset of 13 days (inter-quartile range: 2–59 days; maximal range: 0–13 433 days).

### Drug characteristics

**Fig. 2:**
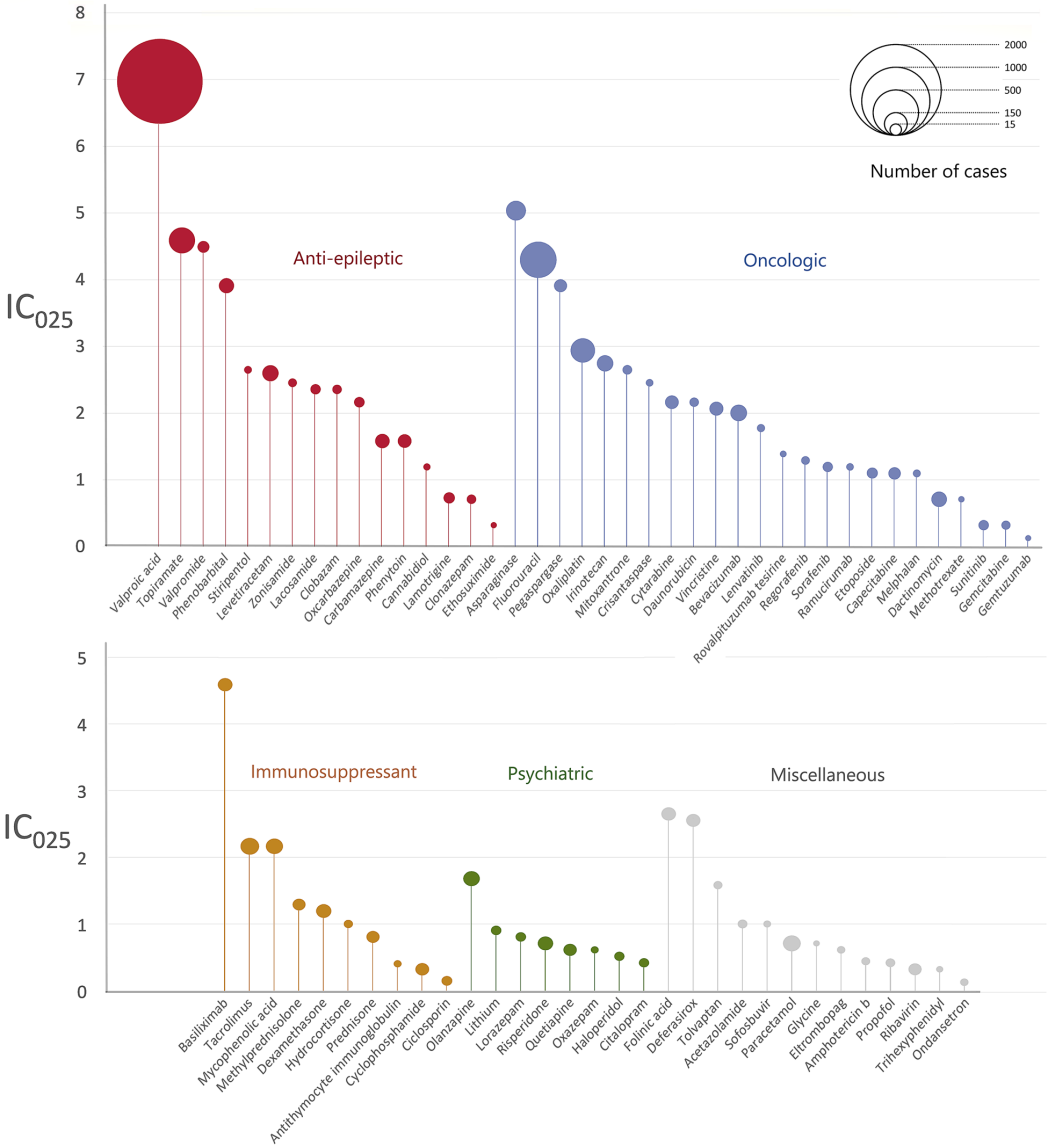
Representation of all suspected liable drugs, as a function of their corresponding IC, IC_025_ and drug class. The size of each dot is proportional to the number of cases in which each drug is suspected involved. The colour code represents the therapeutic class in which each drug belongs: red for anti-epileptics, blue for oncologic agents, yellow for immunosuppressants, green for psychiatric therapeutics and grey for miscellaneous

Most of the suspected drugs (*N* = 58/71, 82%) could be categorised into 4 main therapeutic classes: oncologic drugs (*N* = 24), anti-epileptics (*N* = 16), immunosuppressants (*N* = 10) and psychiatric drugs (*N* = 8). Drugs with the highest number of cases were valproic acid (*N* = 1,722), fluorouracil (*N* = 301), topiramate (*N* = 154), oxaliplatin (*N* = 133) and asparaginase (*N* = 87). Half of the 71 drugs were suspected in over 25 cases of hyperammonaemia. The number of cases associated with each drug is shown in Fig. 2 and Table 1. The magnitude of disproportional association between each drug and hyperammonaemia, identified by IC_025_ values, is shown in Fig. 2 and Table 1. Valproic acid (IC_025_ = 7), asparaginase (IC_025_ = 5.1), topiramate (IC_025_ = 4.7), and basiliximab (IC_025_ = 4.7) were associated with the highest IC_025_. Most drugs were used at a therapeutic level with only 0.2% of (*N* = 7/2924) in suicide attempt circumstances.

**Table 1:** General characteristics per drug associated with hyperammonaemia

Drug	Cases	IC_025_	Age distribution	Median time to onset (days)	Female (%)	Death (%)
Valproic acid	1722	7.0	41.1 (0–93)	9	46.4	3.2
Fluorouracil	301	4.4	64.3 (7–86)	2	33.3	4.3
Topiramate	154	4.7	32.2 (2–90)	16	51.1	0.0
Oxaliplatin	133	3.0	62.6 (7–85)	2	34.6	6.0
Asparaginase	87	5.2	20.7 (2–70)	8	35.8	6.9
Tacrolimus	60	2.2	52.1 (10–72)	122	53.8	56.7
Bevacizumab	58	2.0	62.3 (9–81)	10	27.3	5.2
Irinotecan	56	2.8	60.0 (8–79)	13	35.2	3.6
Levetiracetam	56	2.6	40.7 (2–83)	13	57.7	8.9
Paracetamol	52	0.7	32.5 (2–85)	12	62.7	17.3
Methotrexate	50	0.7	26.4 (2–80)	21	45.8	42.0
Mycophenolic acid	50	2.2	52.0 (15–72)	367	55.8	44.0
Phenobarbital	48	4.0	39.7 (4–78)	14	51.2	4.2
Olanzapine	45	1.7	46.5 (1–88)	17	46.2	0.0
Carbamazepine	43	1.6	37.8 (3–71)	73	48.8	0.0
Risperidone	39	0.7	33.6 (11–81)	17	42.9	2.6
Vincristine	39	2.1	20.1 (2–69)	15	52.8	38.5
Dexamethasone	38	1.2	33.8 (2–81)	2	54.3	44.7
Phenytoin	38	1.6	44.4 (4–81)	13	38.9	5.3
Cytarabine	36	2.2	24.8 (2–70)	24	58.8	61.1
Basiliximab	35	4.7	52.8 (6–72)	NA	56.7	60.0
Folinic acid	35	2.7	55.4 (7–84)	2	35.5	2.9
Deferasirox	34	2.6	14.1 (2–65)	9	61.8	5.9
Pegaspargase	33	4.0	18.9 (2–57)	14	48.3	45.5
Capecitabine	30	1.1	64.3 (24–85)	70	59.3	6.7
Cyclophosphamide	30	0.3	24.6 (2–69)	28	53.6	43.3
Quetiapine	28	0.6	42.7 (13–77)	36	40.7	0.0
Prednisone	27	0.8	40.1 (15–66)	13	52.0	37.0
Ribavirin	27	0.3	48.5 (5–76)	43	29.6	3.7
Valpromide	27	4.6	45.1 (20–68)	7	51.9	0.0
Methylprednisolone	26	1.3	49.1 (16–63)	1	68.4	42.3
Lamotrigine	24	0.7	44.3 (5–79)	49	66.7	12.5
Etoposide	23	1.1	29.2 (2–69)	5	63.6	52.2
Oxcarbazepine	20	2.2	30.9 (4–75)	5	42.1	0.0
Gemcitabine	19	0.3	49.7 (31–76)	8	23.5	0.0
Lacosamide	19	2.4	22.9 (4–65)	3	50.0	15.8
Sorafenib	18	1.2	55.0 (22–70)	20	29.4	11.1
Ciclosporin	16	0.1	27.1 (6–67)	NA	40.0	56.3
Clobazam	15	2.4	32.3 (8–82)	25	42.9	6.7
Clonazepam	15	0.7	37.8 (5–82)	19	64.3	6.7
Lithium	15	0.9	47.5 (11–71)	15	35.7	6.7
Lorazepam	15	0.8	46.9 (17–79)	8	46.2	6.7
Mitoxantrone	15	2.7	29.0 (2–69)	13	53.3	40.0
Citalopram	14	0.4	46.6 (18–83)	4	61.5	0.0
Daunorubicin	14	2.2	13.6 (3–22)	21	69.2	42.9
Haloperidol	14	0.5	37.4 (11–76)	16	58.3	0.0
Sofosbuvir	13	1.0	57.0 (42–76)	46	23.1	7.7
Sunitinib	13	0.3	63.1 (51–78)	14	58.3	15.4
Zonisamide	12	2.5	21.3 (5–58)	220	50.0	8.3
Propofol	11	0.4	47.8 (7–93)	1	20.0	9.1
Regorafenib	11	1.3	59.8 (39–73)	7	0.0	18.2
Hydrocortisone	10	1.0	24.4 (13–66)	9	55.6	70.0
Lenvatinib	10	1.8	67.1 (54–85)	18	20.0	0.0
Tolvaptan	10	1.6	77.8 (60–89)	8	20.0	30.0
Amphotericin b	9	0.4	30.3 (2–82)	15	87.5	22.2
Melphalan	9	1.1	22.4 (2–62)	NA	11.1	22.2
Eltrombopag	8	0.6	32.7 (2–84)	61	62.5	25.0
Ondansetron	8	0.1	19.1 (2–56)	4	12.5	12.5
Stiripentol	8	2.7	8.8 (3–19)	234	75.0	0.0
Crisantaspase	7	2.5	24.8 (3–77)	2	28.6	0.0
Ramucirumab	7	1.2	63.9 (27–80)	35	50.0	0.0
Acetazolamide	6	1.0	65.8 (48–73)	141	33.3	0.0
Antithymocyte immunoglobulin	6	0.4	24.4 (21–45)	NA	55.6	33.3
Cannabidiol	6	1.2	7.3 (5–10)	32	0.0	0.0
Oxazepam	6	0.6	54.8 (41–83)	12	33.3	0.0
Dactinomycin	4	0.7	9.0 (2–23)	38	50.0	25.0
Rovalpituzumab tesirine	4	1.4	49.7 (32–32)	NA	23.5	0.0
Trihexyphenidyl	4	0.3	64.3 (48–80)	40	50.0	0.0
Ethosuximide	3	0.3	10.0 (9–11)	0	0.0	0.0
Gemtuzumab	3	0.1	14.5 (13–16)	23	100.0	33.3
Glycine	3	0.7	54.0 (32–88)	0	66.7	0.0

Cases represents the number of reported cases of each drug, IC_025_ the information component. Age is given in years (means, maximal range) at onset of hyperammonaemia. The time to onset column represents the median time to onset between the introduction of the drug and the occurrence of hyperammonaemia. The female column indicates the proportion of females in cases of hyperammonaemia for each drug studied. Death represents the percentage of death according to each drug. Numbers available to compute those data can be found in Additional file 2: Table S2

NA, not available; IC, information component

### Imputability assessment

The proportion of cases in which no other drug was suspected, the mean number of drugs suspected, the percentages of drug interruption, the percentage of liver dysfunction or kidney dysfunction also then the informativity score and the extrinsic imputability are detailed for each drug in Table 2. Briefly, drugs most often found as the only suspected molecule (i.e. with the signal less confounded by concomitant co-prescriptions) were valpromide (*N* = 19 out of 27 cases including only one suspected drug, 70% of cases) and valproic acid (*N* = 1143/1722, 66%) for anti-epileptics; sunitinib (*N* = 12/13, 92%), sorafenib (*N* = 14/18, 78%), asparaginase (*N* = 56/87, 64%) and regorafenib (*N* = 7/11, 64%) for oncologic drugs; ciclosporin (*N* = 3/16, 19%) and tacrolimus (N = 11/60, 18%) for immunosuppressants; and haloperidol (*N* = 4/14, 29%) for psychiatric drugs. Liver dysfunction was infrequent (*N* = 214/2924, 7.3%) except for dactinomycin (*N* = 4, 100%) and paracetamol (*N* = 34, 65.4%). Kidney dysfunction was also rare (*N* = 86/2924, 3%), except for gemtuzumab (*N* = 1, 33%) and deferasirox (*N* = 10, 32).

**Table 2:** Analysis of potential confounding factors

Drug	Single suspect cases (%)	Mean number of suspect drugs	Drug interruption (%)	Liver dysfunction (%)	Kidney dysfunction (%)	Informativity score NI0 (%)	Extrinsic imputability
Glycine (*N* = 3)	100.0	1.0	0.0	0.0	0.0	66.7	B2
Sunitinib (*N* = 13)	92.3	1.2	53.8	23.1	7.7	30.8	B2
Sorafenib (*N* = 18)	77.8	1.8	27.8	0.0	0.0	22.2	B2
Deferasirox (*N* = 34)	73.5	1.4	8.8	38.2	32.4	73.5	B4
Valpromide (*N* = 27)	70.4	1.4	37.0	3.7	0.0	3.7	B4
Valproic acid (*N* = 1722)	66.4	1.7	42.6	4.9	1.0	28.2	B4
Asparaginase (*N* = 87)	64.4	2.3	54.0	12.6	0.0	6.9	B4
Regorafenib (*N* = 11)	63.6	1.5	18.2	18.2	0.0	18.2	B2
Eltrombopag (*N* = 8)	62.5	1.8	37.5	37.5	0.0	0.0	B1
Crisantaspase (N = 7)	57.1	1.9	14.3	0.0	0.0	0.0	B2
Acetazolamide (*N* = 6)	50.0	1.8	66.7	0.0	0.0	16.7	B2
Zonisamide (*N* = 12)	50.0	6.8	25.0	16.7	8.3	50.0	B4
Paracetamol (*N* = 52)	48.1	2.6	21.2	65.4	23.1	19.2	B2
Amphotericin b (*N* = 9)	44.4	2.9	77.8	11.1	0.0	0.0	B4
Cannabidiol (*N *= 6)	33.3	2.7	33.3	33.3	0.0	33.3	B1
Carbamazepine (*N* = 43)	32.6	3.4	37.2	7.0	0.0	34.9	B3
Pegaspargase (*N* = 33)	30.3	3.3	3.0	33.3	9.1	42.4	B4
Capecitabine (*N* = 30)	30.0	2.8	63.3	3.3	0.0	16.7	B2
Tolvaptan (*N* = 10)	30.0	3.2	50.0	20.0	10.0	20.0	B1
Haloperidol (*N* = 14)	28.6	4.3	0.0	28.6	7.1	50.0	B4
Ramucirumab (*N* = 7)	28.6	3.4	0.0	14.3	0.0	42.9	B2
Dactinomycin (*N* = 4)	25.0	2.5	0.0	100.0	25.0	0.0	B2
Lamotrigine (*N* = 24)	25.0	3.1	58.3	8.3	0.0	29.2	B3
Fluorouracil (*N* = 301)	21.3	3.5	35.9	1.0	4.3	20.3	B4
Lenvatinib (*N* = 10)	20.0	2.7	60.0	10.0	0.0	10.0	B3
Ciclosporin (*N* = 16)	18.8	4.4	18.8	12.5	18.8	37.5	B1
Tacrolimus (*N* = 60)	18.3%	4.3	30.0	3.3	3.3	66.7	B2
Gemcitabine (*N* = 19)	15.8	2.2	15.8	10.5	0.0	36.8	B2
Olanzapine (*N* = 45)	15.6	3.1	20.0	11.1	2.2	48.9	B2
Levetiracetam (*N* = 56)	14.3	5.5	42.9	5.4	5.4	26.8	B2
Topiramate (*N* = 154)	14.3	3.5	29.9	4.5	1.3	46.1	B4
Bevacizumab (*N* = 58)	13.8	4.4	55.2	5.2	1.7	17.2	B2
Lorazepam (*N* = 15)	13.3	5.7	26.7	20.0	0.0	33.3	B2
Phenytoin (*N* = 38)	13.2	4.7	42.1	5.3	2.6	42.1	B3
Stiripentol (*N* = 8)	12.5	5.5	37.5	12.5	12.5	25.0	B2
Lacosamide (*N* = 19)	10.5	4.2	57.9	5.3	5.3	21.1	B2
Hydrocortisone (*N* = 10)	10.0	5.6	20.0	10.0	0.0	30.0	B1
Oxcarbazepine (*N* = 20)	10.0	3.4	55.0	10.0	0.0	35.0	B2
Risperidone (*N* = 39)	7.7	3.7	43.6	2.6	0.0	28.2	B2
Sofosbuvir (*N* = 13)	7.7	2.0	53.8	7.7	15.4	38.5	B2
Prednisone (*N* = 27)	7.4	5.8	37.0	11.1	11.1	33.3	B1
Citalopram (*N* = 14)	7.1	3.6	42.9	14.3	0.0	50.0	B1
Quetiapine (*N* = 28)	7.1	3.2	39.3	10.7	7.1	21.4	B2
Clonazepam (*N* = 15)	6.7	7.5	0.0	13.3	0.0	73.3	B2
Cyclophosphamide (*N* = 30)	6.7	4.4	13.3	23.3	3.3	40.0	B2
Lithium (*N* = 15)	6.7	4.4	13.3	0.0%	6.7	60.0	B2
Irinotecan (*N* = 56)	5.4	5.4	35.7	1.8%	5.4	32.1	B2
Methotrexate (*N* = 50)	4.0	5.0	20.0	34.0	14.0	40.0	B2
Oxaliplatin (*N* = 133)	3.0	4.5	28.6	2.3	3.8	27.1	B2
Folinic acid (*N* = 35)	2.9	5.4	28.6	2.9	8.6	40.0	B2
Vincristine (*N* = 39)	2.6	4.9	15.4	28.2	7.7	41.0	B2
Mycophenolic acid (*N* = 50)	2.0	4.8	20.0	0.0	8.0	52.0	B2
Antithymocyte immunoglobulin (*N* = 6)	0.0	6.0	50.0	0.0	16.7	33.3	B1
Basiliximab (*N* = 35)	0.0	5.1	54.3	0.0	0.0	22.9	B2
Clobazam (*N* = 15)	0.0	5.3	86.7	0.0	0.0	13.3	B3
Cytarabine (*N* = 36)	0.0	4.7	11.1	33.3	5.6	47.2	B2
Daunorubicin (*N* = 14)	0.0	5.0	14.3	50.0	7.1	50.0	B2
Dexamethasone (*N* = 38)	0.0	4.5	31.6	13.2	2.6	57.9	B1
Ethosuximide (*N* = 3)	0.0	3.3	33.3	0.0	0.0	33.3	B2
Etoposide (*N* = 23)	0.0	5.0	56.5	13.0	8.7	34.8	B2
Gemtuzumab (*N* = 3)	0.0	3.0	0.0	33.3	33.3	33.3	B2
Melphalan (*N* = 9)	0.0	4.8	22.2	0.0	22.2	33.3	B2
Methylprednisolone (*N* = 26)	0.0	6.0	42.3	3.8	0.0	50.0	B2
Mitoxantrone (*N* = 15)	0.0	4.5	40.0	6.7	6.7	53.3	B2
Ondansetron (*N* = 8)	0.0	5.3	87.5	25.0	12.5	0.0	B2
Oxazepam (*N* = 6)	0.0	4.3	100.0	33.3	0.0	0.0	B1
Phenobarbital (*N* = 48)	0.0	4.6	41.7%	4.2	4.2	22.9	B3
Propofol (*N* = 11)	0.0	4.8	36.4	18.2	0.0	27.3	B2
Ribavirin (*N* = 27)	0.0	3.3	22.2	14.8	7.4	40.7	B2
Rovalpituzumab tesirine (*N* = 4)	0.0	2.3	0.0	0.0	0.0	100.0	B2
Trihexyphenidyl (*N* = 4)	0.0	3.5	0.0	0.0	0.0	75.0	B2

In this table are represented for each drug the percentage of cases where a single drug was suspected, the mean number of suspect drugs per case, the percentage of interruption of the suspect drug, the percentage of liver and kidney dysfunction, percentage of informativity score NI0 and the extrinsic imputability. NI0 corresponds to absence of both time to onset and discontinuation. For the extrinsic imputability, B4 is quoted when the effect is expected (described in the summary of product characteristics), B3 if the effect is widely published with this drug in reference databases and/or books, B2 if cases are published in a scientific journal, B1, if the effect is not published

Only ten drugs were already described in those labels as being associated with hyperammonaemia in the FDA’s and EMA’s labels (B4 extrinsic imputability): valproic acid, valpromide, topiramate, asparaginase, fluorouracil, haloperidol, pegaspargase, zonisamide, deferasirox and amphotericin B. The frequency of hyperammonaemia is reported as uncommon (between 1/100 and 1/1000 cases) for asparaginase, rare (between 1/1000 and 1/10 000 cases) for valproic acid, valpromide, topiramate and undetermined for the other drugs of this group. Out of the 61 other drugs, 6 have been widely published in reference databases and/or books (B3 extrinsic imputability), 45 have been described in case-reports or case series (B2 extrinsic imputability) and 10 have never been published (B1 extrinsic imputability).

### Clinical characteristics, presentation, and outcome

**Fig. 3:**
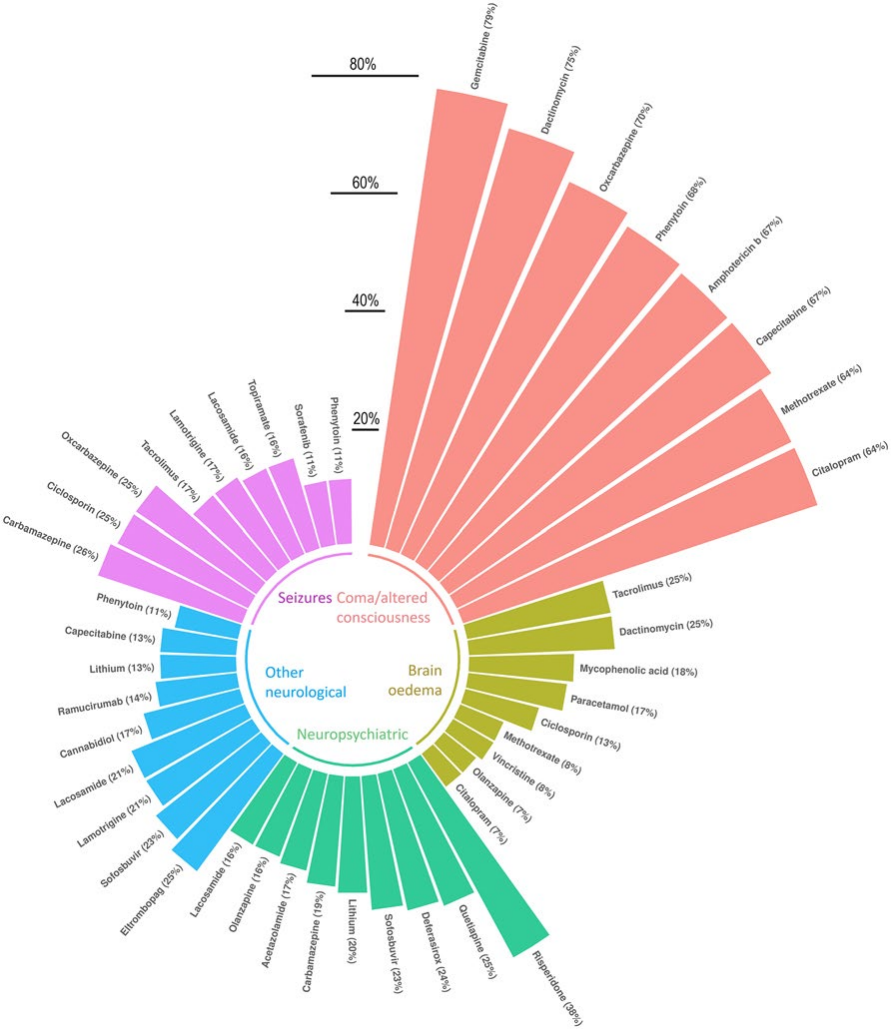
Clinical presentation for each drug: coma/altered consciousness, neuropsychiatric, seizures, brain oedema, other neurological. Results are expressed as a percentage of the total number of cases. Drugs for which causality was difficult to establish (mean number of suspect/interacting drugs of 5 or more, N = 28/71 drugs) were not represented

Detailed demographic characteristics (sex, age, time to onset) for each drug are shown in Table 1. The details of the spectrum of the main clinical presentations for each liable drug are presented in Fig. 3. Gemcitabine (*N* = 15, 79%) and dactinomycin (*N* = 3, 75%) were most often presenting as coma/altered consciousness; carbamazepine (*N* = 11, 26%) and ciclosporin (*N* = 4, 25%) with seizures; risperidone (*N* = 15, 38%) and quetiapine (*N* = 7, 25%) with neuropsychiatric symptoms; tacrolimus (*N* = 15, 25%) and dactinomycin (*N* = 1, 25%) with brain oedema; while eltrombopag (*N* = 2, 25%) and sofosbuvir (*N* = 3, 23%) were mostly presenting with other neurological symptoms.

**Fig. 4:**
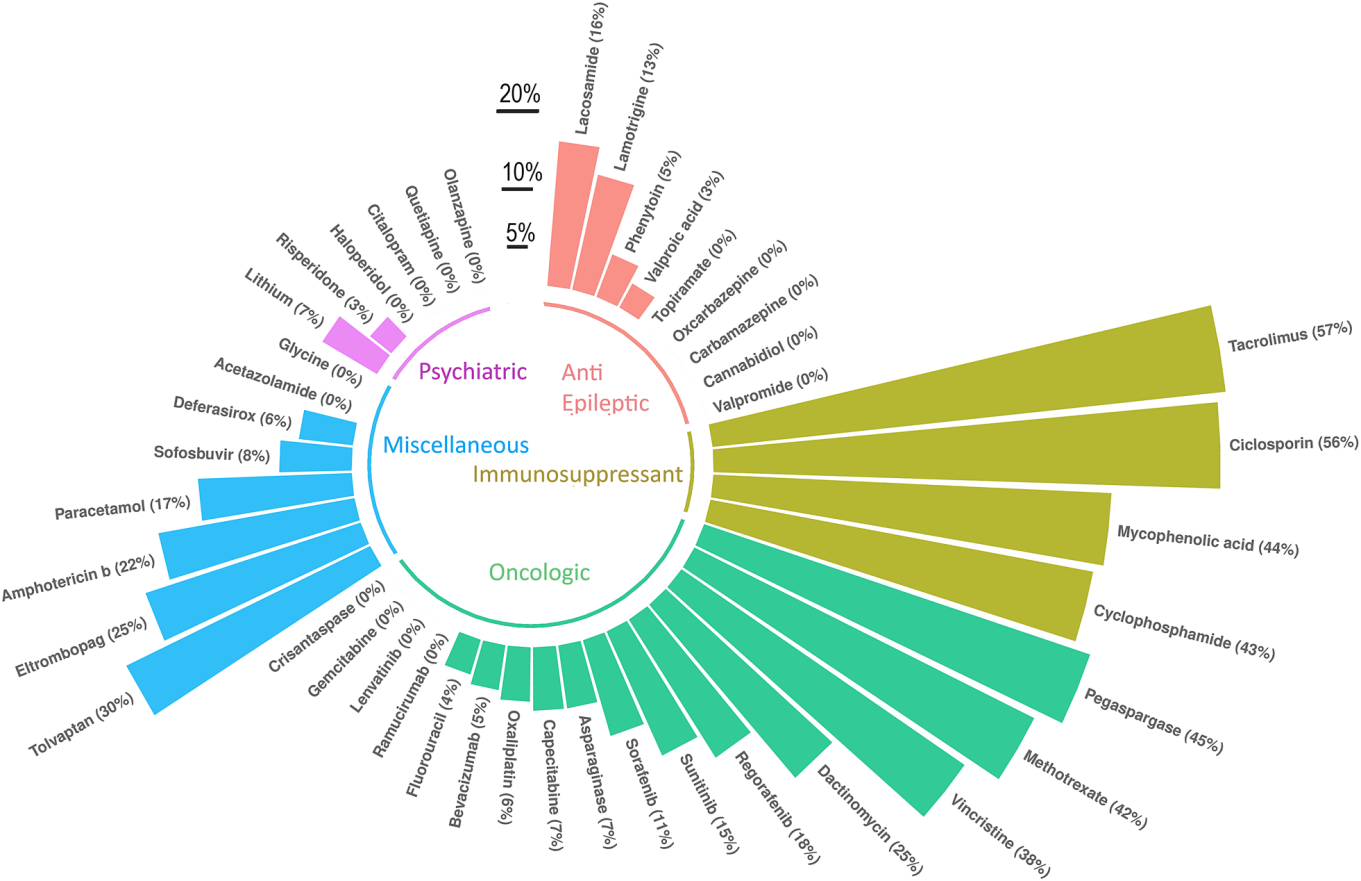
Overall mortality rate associated with suspected drug-associated hyperammonaemia cases. Colour code indicates the therapeutic class: green for oncologic drugs, yellow for immunosuppressants, red for anti-epileptics, purple for psychiatric agent and blue for miscellaneous. Drugs for which causality was difficult to establish (mean number of suspect/interacting drugs of 5 or more, *N* = 28/71 drugs) were not represented

Among the different drugs, the highest mortality rates were found in cases involving tacrolimus (*N* = 34, 57%), ciclosporin (*N* = 9, 56%), pegaspargase (*N* = 15, 45%), mycophenolic acid (*N* = 22, 44%) and cyclophosphamide (*N* = 13, 43%). Details concerning the all-cause mortality rates for all culprit drugs are shown in Table 1 and Fig. 4. The details for the severity of each case and all outcomes (death, life threatening, caused/prolonged hospitalisation, disabling/incapacitating, congenital anomaly/birth defect, other medically important condition) are detailed in Additional file 3: Table S3.

## Discussion

This study constitutes the largest study to date on drug-associated hyperammonaemia and the first description of clinical features and severity for each potential liable drug. Most of the suspected drugs could be categorised into 4 main therapeutic classes: oncologic drugs, anti-epileptic drugs, immunosuppressants and psychiatric drugs. The drugs most frequently involved were valproic acid, fluorouracil, topiramate, oxaliplatin and asparaginase. In addition to these molecules known to be responsible for hyperammonaemia, our study reported 10 drugs not previously identified as responsible for hyperammonaemia (B1 extrinsic imputability). These include recently marketed molecules including anti-epileptics such as cannabidiol, immunosuppressants such as basiliximab, and anti-angiogenics agents such as tyrosine kinase inhibitors (sunitinib, sorafenib, regorafenib, lenvatinib) and monoclonal antibodies (bevacizumab, ramucirumab) [25]. Most reported cases were reported in the last 10 years. Several factors could explain this: first, the number of countries participating in VigiBase increases over time and so do the reports, second, many drugs have been released very recently in the field of oncology and finally, hyperammonaemia could be easily missed if the dosage is not performed.

It should be noted that hyperammonaemia secondary to immunosuppressants and oncological treatments were associated with the highest overall mortality rates. Hyperammonaemia secondary to anti-epileptic and psychiatric drugs had more favourable outcomes. This difference of outcome could be partly driven by more severe underlying comorbidities in patients with immunosuppressants and oncological treatments and the effect of the drugs themselves on mortality needs to be further characterised. Because of the absence of large series in the literature, this could not be compared to previous data, but these results seem to be in accord with the published case reports [26, 27]. It should be noticed that drugs known to be associated with hyperammonaemia such as asparaginase, 5-fluorouracil or capecitabine had lower mortality rates (6.9%, 4.3% and 6.7%, respectively) compared to more recently marketed molecules such as sunitinib, regorafenib or basiliximab (15.4%, 18.2% and 60%, respectively). This could suggest that the identification of the situation and its prompt management might improve the outcome and/or may indicate an awareness bias (i.e. serious cases and those involving new drugs are more likely to be reported). Tacrolimus is one of the drugs responsible for the most cases quantitatively of suspected drug-associated hyperammonaemia in VigiBase (6th out of 71) despite the absence of its mention in FDA and EMA’s drug labels. These latter cases are associated with a 57% mortality, but with data available in VigiBase, determining the exact cause of death in each individual case might be challenging, due to several limitations detailed below including exhaustivity of data information reported in records.

Clinical signs of hyperammonaemia are varied, but the most common presentation was coma or altered state of consciousness. Revealing symptoms could nevertheless be influenced by the underlying conditions of each patient and vary depending on the drug class involved. Indeed, neuropsychiatric symptoms were frequent for patients with psychiatric treatments and seizures often occurred in patients with anti-epileptic treatments. In patients taking immunosuppressants, hyperammonaemia was frequently accompanied by brain oedema or seizures. Time to onset of hyperammonaemia was not limited to the introduction period in the first few days following the liable drug start and could be delayed. Several mechanisms might explain this delayed reaction that has also been found in previous studies [16, 28 ,29]. First, it cannot be ruled out that hyperammonaemia is a dose-dependent adverse effect occurring at a "supratherapeutic" cumulative dose for some medications such as chemotherapies. Furthermore, a modification of the drug’s dose might also increase the risk of hyperammonaemia. Also, many intercurrent events (infection, acute renal failure, liver failure or the instauration of another drug with potential interactions, etc.) might cause hyperammonaemia in a patient taking one of the described drugs. For these reasons, when clinicians are faced with hyperammonaemia and after having excluded classical causes, a drug-associated origin should be suspected. The imputability of each drug should be assessed, based on an evaluation from the pharmacovigilance centre.

The main limitations of our analysis are those of retrospective pharmacovigilance studies from databases. Indeed, current post-marketing pharmacovigilance is strongly based on spontaneous notification and presents well‐known bias [21, 30]. Among these, the main bias concerns the lack of information and the under-notification. For instance, the number of cases with liver failure is strictly low and does not represent the prevalence of liver injuries in the population. This is related to the fact that hyperammonaemia is attributed—maybe wrongly—to the liver disease by hepatologists, who disregard the possibility of a drug-associated event. Due to its international nature, this database is particularly suitable for the identification of rare adverse events such as hyperammonaemia, but those results should be always confirmed by translational studies identifying the mechanisms at play [31, 32]. Moreover, underlying conditions such as cancer or auto-immune disease could also increase the risk of hyperammonaemia and constitute a potential bias. To address these issues and potential biases, we used a comprehensive approach using the informativity score and the extrinsic imputability score classically used in the French pharmacovigilance methodology of causality assessment [24]. Other methods, especially the Naranjo’s developed in Toronto would have been an alternative, but this method is designed for controlled trials rather than clinical routine practice. Furthermore, their uses are almost impossible here in view of the heterogeneity of the recordings and the available information (especially because of the absence of narratives). Finally, analyses on VigiBase do not systematically contain the exact values of lab results (such as ammonaemia or liver enzymes that can be absent or given as values relative to upper normal range), so interpretation of those parameters can only be qualitative. Furthermore, it is important to note that the imputability of each molecule is difficult to assess for drugs that are rarely prescribed in monotherapy, such as some chemotherapies or anti-epileptics. Furthermore, without data on numbers of exposed patients in VigiBase, this work cannot assess the incidence or risk of hyperammonaemia with these drugs. Finally, the description of the clinical presentation during hyperammonaemia should only be considered as exploratory because of the strong influence of the underlying conditions and the difficulty to attribute those symptoms to hyperammonaemia.

## Conclusion

This study constitutes the first large-scale investigation of suspected drug-associated hyperammonaemia, a serious, under-diagnosed and treatable condition, frequent in ICU. Oncologic, anti-epileptic, immunosuppressants and psychiatric drugs are the main therapeutic classes associated with hyperammonaemia. The drugs most frequently involved were valproic acid, fluorouracil, topiramate, oxaliplatin and asparaginase. This description may prove to be useful for clinicians in patients’ care as well as trial design. The supplied data came from a variety of sources.

## Supplementary Information

**Additional file 1: Table S1** MedDRA terms used to constitute each group of adverse events: Coma/altered state of consciousness, brain oedema, seizures, neuropsychiatric presentation, miscellaneous neurological signs, liver dysfunction, kidney dysfunction.

**Additional file 2: Table S2** Missing data for each characteristic: drug role (suspect, interacting or concomitant), number of interactions, sex, age, country, time to onset.

**Additional file 3: Table S3** Outcomes for each drug: Severe case, death, life threatening, caused or prolonged hospitalization, disabling/incapacitating, congenital anomaly/birth defect, other medically important condition. For seriousness criteria, more than one can be chosen.

**Additional file 4: Figure S1** Distribution of cases per year: in abscissa is represented the year of declaration of cases and in ordinate the percentage of all cases.

## Data Availability

The data, analytic methods, and study materials are available to other researchers for purposes of reproducing the results or replicating the procedure at http://www.vigiaccess.org/.
