# Supplementary material for: Drug-associated hyperammonaemia: a Bayesian analysis of the WHO Pharmacovigilance Database
Source: Ann Intensive Care. 2022 Jun 18;12:55. doi: 10.1186/s13613-022-01026-4 (PMC9206694; doi:10.1186/s13613-022-01026-4)
Supplement: Supplementary file 1 — Additional file 1: Table S1 MedDRA terms used to constitute each group of adverse events: Coma/altered state of consciousness, brain oedema, seizures, neuropsychiatric presentation, miscellaneous neurological signs, liver dysfunction, kidney dysfunction. [file 13613_2022_1026_MOESM1_ESM.pdf]

Group of adverse events	MedDRA prefered terms
Coma/altered state of consciousness	Altered state of consciousness, Loss of consciousness, Encephalopathy, Loss of consciousness, Depressed level of consciousness, Encephalopathy, Unresponsive to stimuli, Coma, Lethargy, Mental status changes, Somnolence, Stupor, Hepatic encephalopathy, Hyperammoniemic encephalopathy, Delirium, Confusional state
Brain oedema	Brain death, Brain herniation, Brain injury, Brain oedema, Brain scan abnormal, Brain stem syndrome, Intracranial pressure increased
Seizures	Clonus, Epilepsy, Generalised tonic-clonic seizure, Petit mal epilepsy, Seizure, Status epilepticus
Neuropsychiatric presentation	Personality change, Psychotic disorder, Depression, Delusion, Aggression, Abnormal behaviour, Agitation, Amnesia, Anxiety, Apathy, Behaviour disorder, Cognitive disorder, Dementia, Disinhibition, Disorganised speech, Hallucination, Insomnia, Irritability, Mania, Memory impairment, Mental disorder, Mental impairment, Disorientation, Disturbance in attention
Miscallenous neurological signs	Ataxia, Aphasia, Chorea, Choreoathetosis, Dysarthria, Dyskinesia, Dysphagia, Gait disturbance, Movement disorder, Myoclonus, Tremor
Liver dysfunction	Hepatic failure, Hepatic function abnormal, Hepatic necrosis, Hepatitis, Hepatitis fulminant, Hepatocellular injury, Hepatomegaly, Hepatosplenomegaly, Hepatotoxicity, Transaminases increased, Acute hepatic failure, Autoimmune hepatitis, Bilirubin conjugated increased, Blood bilirubin increased, Cholelithiasis, Cholestasis, Chronic hepatic failure, Chronic hepatitis, Gamma-glutamyltransferase increased, Hepatic cirrhosis, Jaundice, Liver disorder, Liver function test abnormal, Liver injury, Portal hypertension, Alanine aminotransferase abnormal, Alanine aminotransferase increased, Aspartate aminotransferase increased, Aspartate aminotransferase abnormal
Kidney dysfunction	Renal impairment, Renal tubular disorder, Acute kidney injury, Blood creatinine increased, Blood urea increased, Blood uric acid increased, Chronic kidney disease, Renal disorder, Renal failure, Creatinine renal clearance decreased, Dialysis
