# Supplementary material for: Drug-associated hyperammonaemia: a Bayesian analysis of the WHO Pharmacovigilance Database
Source: Ann Intensive Care. 2022 Jun 18;12:55. doi: 10.1186/s13613-022-01026-4 (PMC9206694; doi:10.1186/s13613-022-01026-4)
Supplement: Supplementary file 2 — Additional file 2: Table S2 Missing data for each characteristic: drug role (suspect, interacting or concomitant), number of interactions, sex, age, country, time to onset. [file 13613_2022_1026_MOESM2_ESM.pdf]

Drug	Missing drug role	Missing number of interractions	Missing sex	Missing age	Missing country	Missing time to onset
Acetazolamide	0,0%	0,0%	0,0%	16,7%	0,0%	66,67%
Amphotericin b	0,0%	0,0%	11,1%	33,3%	0,0%	0,00%
Antithymocyte immunoglobulin	0,0%	0,0%	10,0%	10,0%	0,0%	100,00%
Asparaginase	0,0%	0,0%	6,9%	11,5%	0,0%	55,17%
Basiliximab	0,0%	0,0%	14,3%	14,3%	0,0%	100,00%
Bevacizumab	0,0%	0,0%	5,2%	8,6%	0,0%	24,14%
Cannabidiol	0,0%	0,0%	66,7%	50,0%	0,0%	66,67%
Capecitabine	0,0%	0,0%	10,0%	10,0%	0,0%	20,00%
Carbamazepine	0,0%	0,0%	0,0%	2,3%	0,0%	55,81%
Ciclosporin	0,0%	0,0%	6,3%	12,5%	0,0%	100,00%
Citalopram	0,0%	0,0%	7,1%	14,3%	0,0%	71,43%
Clobazam	0,0%	0,0%	6,7%	13,3%	0,0%	13,33%
Clonazepam	0,0%	0,0%	6,7%	6,7%	0,0%	0,00%
Crisantaspase	0,0%	0,0%	0,0%	14,3%	0,0%	0,00%
Cyclophosphamide	0,0%	0,0%	6,7%	20,0%	0,0%	70,00%
Cytarabine	0,0%	0,0%	5,6%	11,1%	0,0%	77,78%
Dactinomycin	0,0%	0,0%	0,0%	25,0%	0,0%	0,00%
Daunorubicin	0,0%	0,0%	7,1%	7,1%	0,0%	28,57%
Deferasirox	0,0%	0,0%	0,0%	5,9%	0,0%	82,35%
Dexamethasone	0,0%	0,0%	7,9%	15,8%	0,0%	0,00%
Eltrombopag	0,0%	0,0%	0,0%	12,5%	0,0%	50,00%
Ethosuximide	0,0%	0,0%	33,3%	33,3%	0,0%	66,67%
Etoposide	0,0%	0,0%	4,3%	8,7%	0,0%	86,96%
Fluorouracil	0,0%	0,0%	2,3%	4,0%	0,0%	24,58%
Folinic acid	0,0%	0,0%	11,4%	14,3%	0,0%	80,00%
Gemcitabine	0,0%	0,0%	10,5%	15,8%	0,0%	63,16%
Gemtuzumab	0,0%	0,0%	0,0%	33,3%	0,0%	66,67%
Glycine	0,0%	0,0%	0,0%	0,0%	0,0%	100,00%
Haloperidol	0,0%	0,0%	14,3%	14,3%	0,0%	14,29%
Hydrocortisone	0,0%	0,0%	10,0%	10,0%	0,0%	80,00%
Irinotecan	0,0%	0,0%	3,6%	7,1%	0,0%	39,29%
Lacosamide	0,0%	0,0%	5,3%	10,5%	0,0%	68,42%
Lamotrigine	0,0%	0,0%	0,0%	12,5%	0,0%	50,00%
Lenvatinib	0,0%	0,0%	0,0%	0,0%	0,0%	40,00%
Levetiracetam	0,0%	0,0%	7,1%	8,9%	0,0%	37,50%
Lithium	0,0%	0,0%	6,7%	20,0%	0,0%	0,00%
Lorazepam	0,0%	0,0%	13,3%	6,7%	0,0%	33,33%
Melphalan	0,0%	0,0%	0,0%	22,2%	0,0%	100,00%
Methotrexate	0,0%	0,0%	4,0%	14,0%	0,0%	76,00%
Methylprednisolone	0,0%	0,0%	26,9%	30,8%	0,0%	73,08%
Mitoxantrone	0,0%	0,0%	0,0%	6,7%	0,0%	93,33%
Mycophenolic acid	0,0%	0,0%	14,0%	16,0%	0,0%	96,00%
Olanzapine	0,0%	0,0%	13,3%	22,2%	0,0%	53,33%
Ondansetron	0,0%	0,0%	0,0%	0,0%	0,0%	0,00%
Oxaliplatin	0,0%	0,0%	4,5%	6,8%	0,0%	25,56%
Oxazepam	0,0%	0,0%	0,0%	16,7%	0,0%	0,00%
Oxcarbazepine	0,0%	0,0%	5,0%	25,0%	0,0%	70,00%
Paracetamol	0,0%	0,0%	1,9%	9,6%	0,0%	69,23%
Pegaspargase	0,0%	0,0%	12,1%	36,4%	0,0%	75,76%
Phenobarbital	0,0%	0,0%	10,4%	14,6%	0,0%	43,75%
Phenytoin	0,0%	0,0%	5,3%	5,3%	0,0%	18,42%
Prednisone	0,0%	0,0%	7,4%	11,1%	0,0%	66,67%
Propofol	0,0%	0,0%	9,1%	9,1%	0,0%	90,91%
Quetiapine	0,0%	0,0%	3,6%	7,1%	0,0%	0,00%
Ramucirumab	0,0%	0,0%	14,3%	0,0%	0,0%	57,14%
Regorafenib	0,0%	0,0%	18,2%	18,2%	0,0%	0,00%
Ribavirin	0,0%	0,0%	0,0%	33,3%	0,0%	22,22%
Risperidone	0,0%	0,0%	10,3%	17,9%	0,0%	71,79%
Rovalpituzumab tesirine	0,0%	0,0%	10,5%	15,8%	0,0%	100,00%
Sofosbuvir	0,0%	0,0%	0,0%	30,8%	0,0%	61,54%
Sorafenib	0,0%	0,0%	5,6%	22,2%	0,0%	50,00%
Stiripentol	0,0%	0,0%	0,0%	0,0%	0,0%	12,50%
Sunitinib	0,0%	0,0%	7,7%	30,8%	0,0%	61,54%
Tacrolimus	0,0%	0,0%	13,3%	16,7%	0,0%	96,67%
Tolvaptan	0,0%	0,0%	0,0%	10,0%	0,0%	80,00%
Topiramate	0,0%	0,0%	12,3%	16,9%	0,0%	76,62%
Trihexyphenidyl	0,0%	0,0%	0,0%	0,0%	0,0%	0,00%
Valproic acid	0,0%	0,0%	6,7%	13,9%	0,0%	68,29%
Valpromide	0,0%	0,0%	0,0%	11,1%	0,0%	29,63%
Vincristine	0,0%	0,0%	7,7%	20,5%	0,0%	53,85%
Zonisamide	0,0%	0,0%	16,7%	25,0%	0,0%	41,67%
