# Supplementary material for: Drug-associated hyperammonaemia: a Bayesian analysis of the WHO Pharmacovigilance Database
Source: Ann Intensive Care. 2022 Jun 18;12:55. doi: 10.1186/s13613-022-01026-4 (PMC9206694; doi:10.1186/s13613-022-01026-4)
Supplement: Supplementary file 3 — Additional file 3: Table S3 Outcomes for each drug: Severe case, death, life threatening, caused or prolonged hospitalization, disabling/incapacitating, congenital anomaly/birth defect, other medically important condition. For seriousness criteria, more than one can be chosen. [file 13613_2022_1026_MOESM3_ESM.pdf]

Drug	Serious	Death	Life threatening	Caused/prolonged hospitalization	Disabling/incapacitating	Congenital anomaly/birth defect	Other medically important condition
Acetazolamide	50%	0%	0%	33%	0%	0%	17%
Amphotericin b	56%	22%	0%	11%	0%	0%	33%
Antithymocyte immunoglobulin	100%	33%	17%	33%	0%	0%	67%
Asparaginase	72%	7%	9%	38%	1%	0%	36%
Basiliximab	100%	60%	31%	37%	0%	0%	43%
Bevacizumab	100%	5%	21%	41%	0%	0%	64%
Cannabidiol	83%	0%	0%	67%	0%	0%	17%
Capecitabine	90%	7%	10%	47%	3%	0%	57%
Carbamazepine	74%	0%	5%	44%	0%	0%	47%
Ciclosporin	100%	56%	6%	31%	0%	0%	63%
Citalopram	79%	0%	7%	57%	0%	0%	50%
Clobazam	87%	7%	7%	47%	0%	0%	33%
Clonazepam	93%	7%	13%	60%	0%	0%	40%
Crisantaspase	57%	0%	29%	43%	0%	0%	0%
Cyclophosphamide	73%	43%	3%	37%	0%	0%	23%
Cytarabine	92%	61%	14%	64%	0%	0%	22%
Dactinomycin	25%	25%	0%	0%	0%	0%	0%
Daunorubicin	86%	43%	7%	57%	7%	0%	7%
Deferasirox	94%	6%	47%	68%	3%	0%	29%
Dexamethasone	100%	45%	42%	63%	0%	0%	42%
Eltrombopag	88%	25%	38%	75%	0%	0%	38%
Ethosuximide	67%	0%	0%	0%	0%	0%	67%
Etoposide	83%	52%	4%	52%	0%	0%	26%
Fluorouracil	96%	4%	21%	52%	1%	0%	41%
Folinic acid	100%	3%	26%	60%	0%	0%	69%
Gemcitabine	68%	0%	37%	47%	0%	0%	37%
Gemtuzumab	67%	33%	0%	67%	0%	0%	0%
Glycine	67%	0%	33%	67%	0%	0%	33%
Haloperidol	100%	0%	14%	71%	0%	0%	50%
Hydrocortisone	90%	70%	20%	80%	0%	0%	10%
Irinotecan	96%	4%	25%	57%	0%	0%	48%
Lacosamide	95%	16%	11%	42%	5%	0%	63%
Lamotrigine	79%	13%	17%	42%	0%	0%	50%
Lenvatinib	100%	0%	0%	80%	0%	0%	20%
Levetiracetam	82%	9%	13%	46%	2%	0%	52%
Lithium	87%	7%	13%	60%	0%	0%	53%
Lorazepam	87%	7%	33%	67%	0%	0%	67%
Melphalan	89%	22%	11%	56%	0%	0%	44%
Methotrexate	96%	42%	24%	62%	0%	0%	42%
Methylprednisolone	100%	42%	19%	58%	0%	0%	58%
Mitoxantrone	87%	40%	7%	80%	0%	0%	13%
Mycophenolic acid	98%	44%	26%	42%	0%	0%	50%
Olanzapine	76%	0%	24%	53%	2%	0%	38%
Ondansetron	75%	13%	13%	38%	0%	0%	38%
Oxaliplatin	95%	6%	20%	42%	1%	0%	50%
Oxazepam	100%	0%	33%	100%	0%	0%	17%
Oxcarbazepine	90%	0%	5%	40%	5%	0%	45%
Paracetamol	63%	17%	12%	52%	0%	0%	25%
Pegaspargase	97%	45%	27%	61%	0%	0%	33%
Phenobarbital	85%	4%	13%	54%	4%	0%	52%
Phenytoin	82%	5%	5%	45%	3%	0%	50%
Prednisone	93%	37%	11%	56%	0%	0%	56%
Propofol	100%	9%	9%	64%	0%	0%	73%
Quetiapine	86%	0%	14%	64%	0%	0%	46%
Ramucirumab	100%	0%	14%	86%	0%	0%	29%
Regorafenib	82%	18%	0%	73%	0%	0%	45%
Ribavirin	81%	4%	0%	56%	0%	0%	41%
Risperidone	92%	3%	21%	54%	3%	0%	62%
Rovalpituzumab tesirine	100%	0%	50%	100%	0%	0%	25%
Sofosbuvir	85%	8%	0%	69%	8%	0%	31%
Sorafenib	72%	11%	6%	56%	0%	0%	33%
Stiripentol	88%	0%	13%	38%	13%	0%	50%
Sunitinib	100%	15%	15%	77%	0%	0%	31%
Tacrolimus	97%	57%	22%	43%	0%	0%	48%
Tolvaptan	100%	30%	20%	20%	20%	0%	90%
Topiramate	77%	0%	4%	49%	1%	0%	46%
Trihexyphenidyl	100%	0%	0%	75%	25%	0%	0%
Valproic acid	68%	3%	7%	43%	1%	0%	32%
Valpromide	63%	0%	7%	48%	0%	0%	7%
Vincristine	79%	38%	15%	56%	3%	0%	26%
Zonisamide	83%	8%	17%	42%	8%	0%	42%
