# Supplementary figures and images for: Drug-associated hyperammonaemia: a Bayesian analysis of the WHO Pharmacovigilance Database
Source: Ann Intensive Care. 2022 Jun 18;12:55. doi: 10.1186/s13613-022-01026-4 (PMC9206694; doi:10.1186/s13613-022-01026-4)

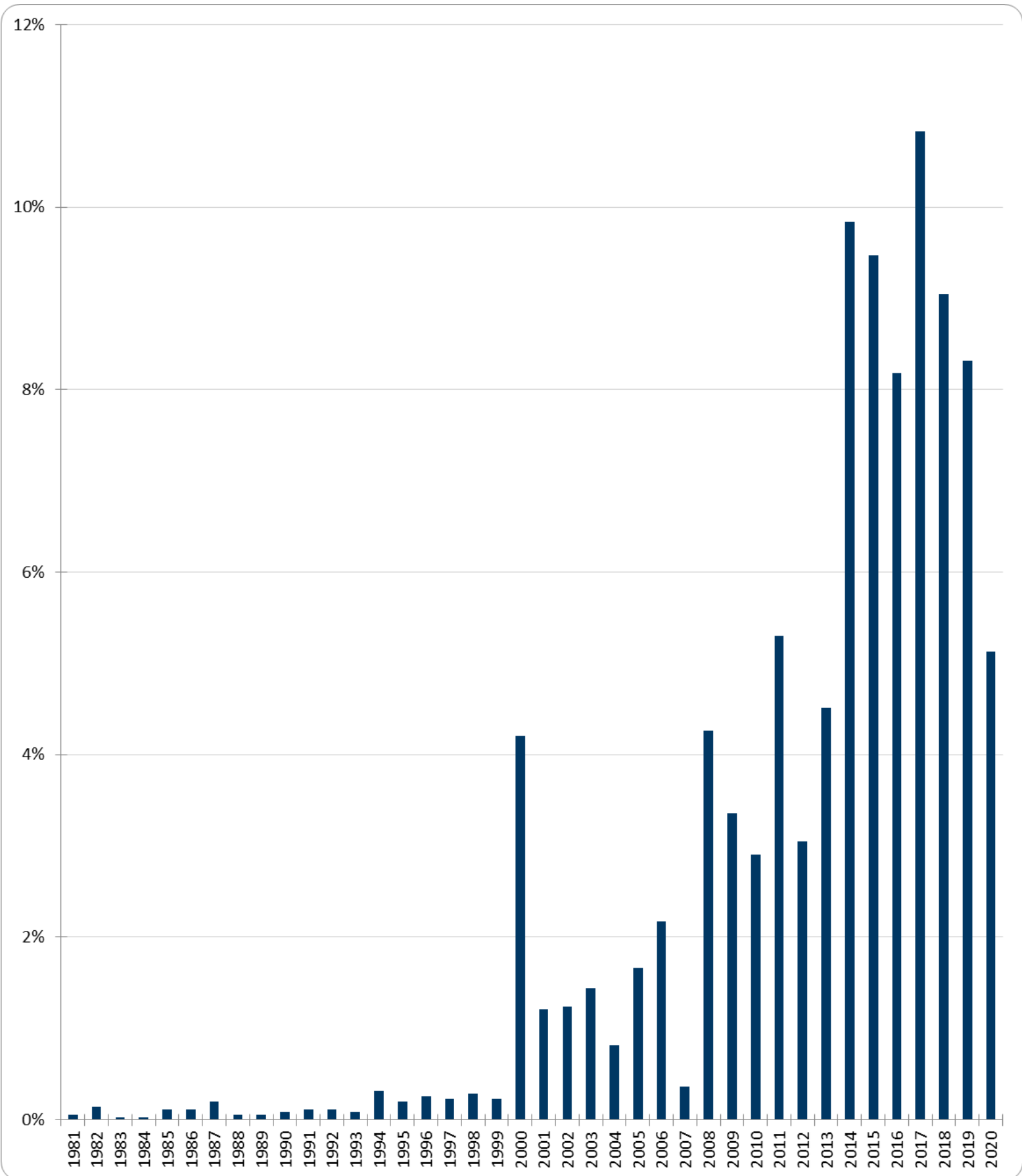

Supplement: Supplementary file 4 — Additional file 4: Figure S1 Distribution of cases per year: in abscissa is represented the year of declaration of cases and in ordinate the percentage of all cases. [file 13613_2022_1026_MOESM4_ESM.pdf]
